# Hospitals and the Rates

**Published:** 1909-09-04

**Authors:** 


### HOSPITALS AND THE RATES.

On August 31, Mr. Jeremiah MacVeagh, M.P., introduced into the House of Commons a Bill empowering local authorities to levy a rate not exceeding threepence in the pound towards the erection and maintenance of hospitals within their area. It is stated that the intention of the promoter is no more than to get the Bill printed this session, the object being to have the matter thoroughly discussed out of doors. Members of all parties are included among those who have promised to "back" the Bill with this end in view.

As this proposal is quite evidently the thin end of a wedge whose driving home means the complete municipalisation of all hospitals, we shall defer comment until next week.

